# Impact of discontinuation of contact precautions on surveillance- and whole-genome sequencing-defined MRSA infections

**DOI:** 10.1017/ash.2023.227

**Published:** 2023-09-29

**Authors:** Sharon Karunakaran, Lora Pless, Ashley Ayres, Carl Ciccone, Joseph Penzelik, Alexander Sundermann, Elise Martin, Marissa Griffith, Kady Waggle, Lee Harrison, Graham Snyder

## Abstract

**Background:** Current guidelines recommend contact precautions to prevent transmission of methicillin-resistant *Staphylococcus aureus* (MRSA) in acute-care hospitals. Prior literature demonstrates that discontinuation of contact precautions for MRSA has not been associated with an increase in carriage rates including surveillance-defined healthcare-associated infection (HAI) while horizontal infection prevention strategies are implemented. **Objective:** To analyze the impact of discontinuation of contact precautions on the rate of MRSA infections, including surveillance-defined HAI and transmission events identified through whole-genome sequencing (WGS) surveillance. **Methods:** In this single tertiary-care center, retrospective, observational, quality improvement analysis, we measured 2 MRSA HAI outcomes before and after discontinuation of contact precautions (ie, gown and gloves no longer required for care of patients with prior or current MRSA infections or colonization, effective December 2, 2020). First, we conducted a time-series analysis using linear regression modelling of NHSN reported MRSA HAI rates (January 2019–November 2022). We also calculated the frequency of WGS-confirmed MRSA transmission events before in the discontinuation of contact precautions (January 2019–August 2019) and after the discontinuation of contact precautions (January 2022–November 2022) periods. Surveillance HAI events were determined using NHSN definitions; MRSA transmission events were defined as an isolate identified ≥3 days after hospitalization or within 30 days of a healthcare exposure, genetically related by ≤15 single-nucleotide polymorphisms compared to ≥1 previously sequenced MRSA isolate. **Results:** We identified 171 MRSA HAIs in the 23 months before discontinuation of contact precautions, corresponding to 4.24 HAI per 10,000 patient days, and 129 HAIs in the 24 months after discontinuation of contact precautions, corresponding to 3.01 HAI per 10,000 patient days (Fig.). We detected a nonsignificant change in the trend in HAI rate before and after discontinuation of contact precautions (*P* = .22) as well as a significant immediate decrease in the MRSA HAI rate (*P* < 0 .001) at the time of discontinuation of contact precautions. In the WGS analysis 8 months before discontinuation of contact precautions, 11 MRSA transmission events were confirmed, comprising 4 clusters (0.75 per 10,000 patient days). In the WGS for the 11-month analysis period after discontinuation of contact precautions, there were 23 confirmed MRSA transmission events comprising 10 clusters (1.22 per 10,000 patient days; incidence rate ratio, 1.61; 95% CI, 0.75–3.66; *P* = .19). **Conclusions:** After discontinuation of contact precautions, there was no significant increase in MRSA HAI or transmission events. Further evaluation of the individual WGS transmission clusters will be helpful to determine whether discontinuation of contact precautions led to MRSA transmission in this facility in the period after discontinuation of contact precautions.

**Figure:**
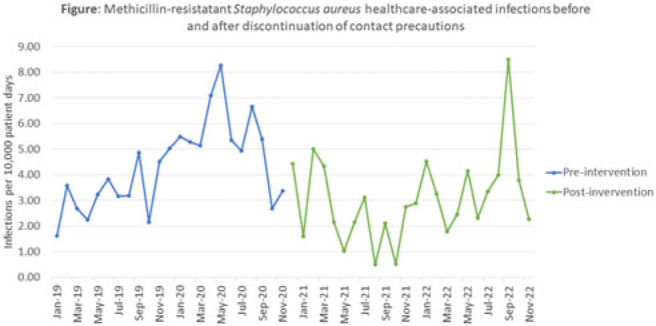

**Disclosure:** None

